# An intelligent referee selection approach in martial arts using CoCoSo MCDM algorithm

**DOI:** 10.1038/s41598-025-08521-1

**Published:** 2025-07-10

**Authors:** Tao Zhang

**Affiliations:** Physical Education, Shaanxi University of Technology, Hanzhong, 723000 Shaanxi China

**Keywords:** CoCoSo algorithm, Decision accuracy, Interval-valued t-spherical fuzzy information, Martial arts, Multi-criteria decision-making, Referee selection, Engineering, Mathematics and computing

## Abstract

Martial arts tournament regulation requires strict adherence to principles which include fairness and both disciplined behavior and accurate decision-making processes. Competition martial arts need referees to uphold integrity by carrying out fair rule enforcement together with exact decision-making responsibilities. The selection process for referees becomes problematic due to unclear evaluation methods, which include decision unpredictability, together with subjective assessment, and qualifications that differ from one another. The research utilizes the CoCoSo multi-criteria decision-making (MCDM) algorithm and interval-valued t-spherical fuzzy sets (IVTSFS) to process referee candidate evaluations with greater accuracy and methodically under uncertain situations. Three expert evaluators conduct assessments of different referee candidates through vital selection parameter analysis, which includes assessment of experience levels, along with decision accuracy rules, understanding physical fitness capabilities, stress management competencies, consistency performance, and communication abilities. Both the CoCoSo algorithm and IVTSFS system enable successful integration of expert judgments and handle ambiguous referee decision-making approaches respectively. The research presents an optimized process for athletic referee selection which guarantees fair competition and selects referees who demonstrate the best qualities during martial arts competitions.

## Introduction

Martial arts competition regulations consist of core standards that enforce fairness, in addition to discipline, precision, and respect to maintain match integrity. The execution of matches by referees depends fundamentally on their role to enforce legal boundaries while assessing player capabilities and taking instant decisions determining the final results. The process of selecting capable referees proves difficult because referees show different levels of experience and decision accuracy and consistency. Traditional selection approaches use subjective judgments to assign referees, but such choices introduce inconsistent and biased evaluations. Multiple key performance factors need a structured, intelligent decision-making approach to evaluate and rank referees during selection procedures properly.

Tournaments in martial arts are usually conducted in a well-formatted manner with different weight classes and divisions, and in many cases, they are performed in rounds. A team of officials, consisting of a central referee, two or three side judges, and a timekeeper, is typically needed per match. A governing committee (e.g., officiating panel or referee committee) typically appoints the officiating team. The appointment of referees is mainly done based on availability, experience, or by manual committee decision, which brings in the element of bias and inconsistency. In major events where dozens of matches are played and hundreds of qualified referees are available, selecting referees fairly and optimally is even more challenging without a systematic assessment method. This paper addresses that requirement by suggesting a knowledge-based, bias-free intelligent referee selection decision model using data.

The appointment of the referees is a critical part of the martial arts competition, since the referees directly impact the fairness, safety, and integrity of matches. Inappropriate selection of referees based on bias, inconsistency, or the absence of formal assessment may result in unfair results, conflicts, and loss of morale among the athletes as well as the reputation of the tournament. In addition, since martial arts involve close contact and high-stakes physical interaction, the referees should show technical mastery and the ability to make decisions in pressuring situations. Regardless, the majority of referee appointments continue to be based upon subjective criteria, or the informal opinions of committees, and these may lack consistency or be subject to internal politics. This presents the strong necessity of a systematic, objective, and transparent referee selection system that considers expert uncertainty and many performance criteria. Thus, the research will solve a real and urgent issue in sport management and refereeing, which is specifically concerned with the national and international tournaments in martial arts.

Human decisions in referee selection create uncertainty because judgment processes are frequently marked by hesitation and imprecision. To handle uncertain conditions, fuzzy sets (FS) were first introduced by Zadeh^1^, which includes membership degrees (MD) for uncertainty modeling. The FS model did not have built-in capabilities to show hesitancy in decision-making. Intuitionistic fuzzy sets (IFS) which Atanassov^2^ developed contain MD and non-membership degree (NMD) as means to address uncertainty within decision-making processes. Yager^3^ developed pythagorean fuzzy sets (PyFS) and q-rung orthopair fuzzy sets (q-ROFS)^4^ both of which improved the capability to handle vague information in decision-making systems. The search for a more expressive framework led Cuong^5^ to develop picture fuzzy sets (PFS) with the addition of an abstention degree (AD) to enable neutral decision-making. spherical fuzzy sets (SFS)^6^ and t-spherical fuzzy sets (T-SFS)^7^ built upon previous advancements to provide enhanced modeling capabilities of uncertainty, thus becoming appropriate tools especially for complex problems like referee selection.

The evaluation process of selecting referees effectively and without bias employs MCDM approaches that are used widely across various sectors, including sports management. Several alternatives received evaluation through MCDM approaches including TOPSIS^8^, VIKOR^9^, SWARA^10^ and PROMETHEE^11^ for handling conflicting criteria. The integration of these approaches proves difficult because they encounter obstacles when dealing with complex relationships within assessment parameters and unclear definitions of expert qualifications. Research requires the CoCoSo algorithm^12^ since it provides a reliable MCDM framework that unifies multiple decision-making components to establish trustworthy rankings. The current research initiative employs IVTSFS^13^ and CoCoSo to develop an assessment framework that increases evaluation accuracy for referee selection even with multiple uncertain parameters. An impartial assessment approach based on seven specified criteria helps the evaluation system to deliver improved officiating standards in martial arts competitions.

### Research gap and motivations

Competitions in martial arts require extensive procedures for selecting referees because these decisions influence both the competition fairness and disciplinary approaches as well as the overall standards of integrity. The present selection procedure bases itself on human evaluation yet heavily depends on standard testing methods, resulting in uneven and favoritism decision outcomes. The MCDM techniques employed for referee selection fail to control expert evaluators’ uncertain behavior and hesitations. Decision scenarios that emerge from uncertain situations find effective solutions through the utilization of various fuzzy-based mathematical models such as IFS, PyFS and PFS. The evaluation methods lack proper mechanisms for detecting human behavioral reactions that develop during referee candidate selection. Research has failed to demonstrate how MCDM capabilities could be merged with sophisticated fuzzy models resulting in poor progress for developing precise selection frameworks. The presented work demonstrates an intelligent CoCoSo MCDM algorithm which links with IVTSFS to develop a system for accurate and reliable referee selection. Experts can make flexible and accurate assessments through IVTSFS, combining MD, NMD, AD, and refusal degree (RD) into an interval structure to handle uncertainty. The CoCoSo algorithm enables experts to efficiently merge their assessments for generating referee rankings through various evaluation standards. The new integration framework presents an orderly assessment method without prejudice, optimizing decision accuracy by reducing human judgment effects.

The goal of this research is to build a trustworthy decision tool that selects better referees for martial arts competitions. Using IVTSFS together with CoCoSo creates in this research a specific selection procedure that employs flexible structured assessment procedures to select referees who satisfy predetermined standards. The model provides increased officiating standards through an authentic decision support system that future research projects can utilize for their selection and evaluation methods.

### Objectives and contributions

The main objectives of this research are summarized below:

- The proposed framework develops an advanced selection process for referees which combines fuzzy logic with MCDM techniques.
- IVTSFS enables superior expert evaluation handling because it provides multiple options to show hesitation levels during the assessment process.
- An aggregating system built with CoCoSo follows expert opinion assessments to determine referee rankings according to multiple essential criteria.

The proposed research makes substantial impacts which benefit both theoretical frameworks and practical applications of these findings. This research studies IVTSFS and CoCoSo for sports decision-making processes because these methods efficiently handle evaluation situations with complex and uncertain factors. Mathematical approaches strengthen MCDM applications in referee selection by providing academic research with a meaningful reference point. This proposed intelligent decision-support system established by the research framework allows organizations and governing bodies to select referees more accurately while achieving transparency in their assignment process. Through this research method, organizations can choose experienced referees who create high-quality officiating while moderating bias to achieve uniform officiating policies in martial arts sports.

### Significance of the study

Research value stems from developing an evaluation framework that utilizes intelligent methods to handle uncertainties throughout referee assessments, leading to data-based systematic processes for selection. The proposed study uses CoCoSo MCDM algorithm in combination with IVTSFS to create an effective solution for decision uncertainty handling within referee rankings based on significant factors. The research establishes new possibilities to strengthen MCDM and fuzzy logic methods in sports management through better uncertainty administration and decision clarity. This investigation has significant implications for sports organizations, competition authorities, and referee selection boards. The developed framework provides a system that mitigates biases, which selects referees through data-driven methods for superior championship refereeing, alongside reduced prejudice in martial arts contests. The study presents an adjustable framework that referees can use in different sports for evaluator assessment due to its core evaluation aspect. The developed method improves referee selection processes, promoting fair competition standards and maintaining overall sport fairness.

### Structure of the study

The rest of the paper is summarized as follows: “Literature review” represents the literature review related to this study. Section “Preliminary knowledge” discusses the preliminaries related to IVTSFS. Section “IVTSF-CoCoSo algorithm” proposed the IVTSF-CoCoSo MCDM algorithm. Section “Assessment of referee selection in martial arts” optimizes the referee selection for martial arts and discusses its results. Section “Comparison analysis” performs a comparison analysis of the proposed algorithm with existing MCDM methods. Section “Sensitivity analysis” performs sensitivity analysis to check the stability of the ranking, also discusses the advantages and limitations of the study. Finally, the study concludes with future research in “Conclusion”.

## Literature review

This section discusses the existing martial arts referee selection studies and CoCoSo MCDM algorithm across multiple fields.

### MCDM in referee selection in martial arts

Multiple existing studies focused on martial arts refereeing decisions and evaluations demonstrate the significance of selecting impartial referees efficiently. Salmhofer^14^ and Salmhofer et al.^15^ used games to create refereeing performance simulation programs for sports officials to improve their decision abilities. The developers from Akhmedzyanov et al.^16^ created an IT-powered Kobudo referee training system that relied on expert scoring for performance evaluations. Channon and Khomutova^17^ analyzed psychological and cognitive requirements that influence mixed martial arts (MMA) referees, and Channon^18^ studied how referees deal with desirable social risks within combat sports. According to Gift^19^, an investigation of MMA judging preferences showed that biased factors play a role in deciding referee outcomes. The reliability and validity of Kumite refereeing in Karate-Do received examination from Fidalgo^20^ who emphasized how well-developed perceptible selection abilities play a crucial role. The study conducted by Hoelbling et al.^21^ showed that video-based training enhanced kickboxing referee judgment accuracy when evaluated against one another.

To reinforce the contextual basis of the suggested framework of referee selection, the literature in the broader sports field has been integrated, and it is concerned with referee appointments, promotion, and decision-making. In professional basketball, Duran et al.^22^ used operations research methods to show how referee assignments can be optimized using computational models to achieve fairness and efficiency in deployment. The study by Webb et al.^23^ focused on referee promotion systems within English football and the importance of such schemes as CORE and their effect on talent identification and career advancement. With neuro-decision science, Zhang et al.^24^ studied how the home advantage affects the referees regarding decision-making, and Kittel et al.^25^ offered a meta-analysis of decision-making training efficacy in sports referees. Hug^26^ emphasized a cognitive load in making referee judgments that combined several evaluation criteria. Though the studies are not aimed at martial arts in particular, they provide essential methodological and theoretical considerations, which facilitate the creation of an effective, data-driven model of referee selection in martial arts competitions. The research field of martial arts refereeing gained valuable insights yet failed to develop any structured MCDM approach for selecting qualified referees. A substantial research gap exists regarding IVTSFS applications for referee evaluation since no previous study has adopted this approach, which our study seeks to address.

### Studies employing CoCoSo MCDM algorithm

The CoCoSo method stands out in MCDM applications because it solves complex decision problems successfully. According to their findings, Yazdani et al.^12^ developed CoCoSo as a combinatory methodology that outperforms conventional MCDM approaches. Research now uses IVTSFS in different domains to extend its usefulness. Qiyas et al.^27^ applied CoCoSo with PFS to boost decision support systems’ performance and Peng and Garg^28^ introduced the conceptual combination of CoCoSo with CRITIC for cache placement technique selection. The decision-making technique proposed by Zheng et al.^29^ relies on interval-valued q-ROFS to enhance CoCoSo’s operation in uncertain contexts. Wang et al.^30^ optimized waste clothing recycling channels by extending CoCoSo using PyFS. The research conducted by Xu et al.^31^ showed how SFS enable CoCoSo to address expert uncertainty better and Ahmad et al.^32^ applied this approach to optimize the oil and gas supply chain logistics. Lai et al.^33^ enhanced CoCoSo aggregation operators for cloud service provider selection while Panchagnula et al.^34^ implemented CoCoSo to optimize cryogenic drilling processes.

CoCoSo and IVTSF models have been previously applied in areas like medical diagnosis^35,36^, human resources recruitment^37^, and supply chain design^38,39^, where multicriteria decisions have to be made under uncertainty and are interrelated. Likewise, the subjectivity and uncertainty of referee selection in the martial arts are also huge matters of concern, as various variables need to be balanced, including experience, correctness of decisions, physical condition, and stress resistance. Based on this parallel, the present study applies the CoCoSo-IVTSF model to the sports context, showing that it can be easily transferred across different realms and assist with making structured decisions that are less biased and have larger error tolerances. Research lacks an application of the CoCoSo method for choosing referees in martial arts competitions under IVTSFS despite its wide range of uses. The study makes a unique contribution by filling a noticeable research gap regarding this problem.

## Preliminary knowledge

This section outlines the preliminary context and mathematical expressions required to grasp the proposed framework. These preliminaries incorporate IVTSFS into the overall process of the CoCoSo method in resolving referee selection in martial arts.

### Definition 1

^7^ Let$\:\:U$ is the discourse universe that includes MD $\:\left(m\right)$, AD $\:\left(a\right)$, and NMD $\:\left(n\right)$. The technical definition of TSFS °F is:

$${}^{\circ}F=\left\{\left({m}_{{}^{\circ}F}\left(u\right),{a}_{{}^{\circ}F}\left(u\right),{n}_{{}^{\circ}F}\left(u\right)\right):u\in\:u\right\}$$

where

$$\:{m}_{{}^{\circ}F }\left(u\right),{a}_{{}^{\circ}F }\left(u\right),{n}_{{}^{\circ}F }\left(u\right)\in\:\left[\text{0,1}\right]$$.

Under these conditions:

1 $$\:0\le\:{\left({m}_{{}^{\circ}F }\left(u\right)\right)}^{t}+{\left({a}_{{}^{\circ}F }\left(u\right)\right)}^{t}+{\left({n}_{{}^{\circ}F }\left(u\right)\right)}^{t}\le\:1\:$$

Furthermore, the RD is well-known by:

$$\:{\pi\:}_{{}^{\circ}F }\left(u\right)={\left(1-\left({\left({m}_{{}^{\circ}F }\left(u\right)\right)}^{t}+{\left({a}_{{}^{\circ}F }\left(u\right)\right)}^{t}+{\left({n}_{{}^{\circ}F }\left(u\right)\right)}^{t}\right)\right)}^{\frac{1}{t}}$$

### Definition 2

^13^ Let $\:U$ is the discourse universe that includes MD$\:\:\left(m\right)$, AD$\:\:\left(a\right)$, and NMD$\:\:\left(n\right)$. The technical definition of IVTSFS $\:{}^{\circ}F$ is:

$$\:{}^{\circ}F =\left\{\left([{m}_{{}^{\circ}F }{\left(u\right)}^{l},{m}_{{}^{\circ}F }{\left(u\right)}^{u}],[{a}_{{}^{\circ}F }{\left(u\right)}^{l},{a}_{{}^{\circ}F }{\left(u\right)}^{u}],[{n}_{{}^{\circ}F }{\left(u\right)}^{l},{n}_{{}^{\circ}F }{\left(u\right)}^{u}]\right):u\in\:u\right\}$$

where:

$$\:[{m}_{{}^{\circ}F }{\left(u\right)}^{l},{m}_{{}^{\circ}F }{\left(u\right)}^{u}],[{a}_{{}^{\circ}F }{\left(u\right)}^{l},{a}_{{}^{\circ}F }{\left(u\right)}^{u}],[{n}_{{}^{\circ}F }{\left(u\right)}^{l},{n}_{{}^{\circ}F }{\left(u\right)}^{u}]\in\:\left[\text{0,1}\right]$$

Under these conditions:

2 $$\:0\le\:{\left({m}_{{}^{\circ}F }{\left(u\right)}^{u}\right)}^{t}+{\left({a}_{{}^{\circ}F }{\left(u\right)}^{u}\right)}^{t}+{\left({n}_{{}^{\circ}F }{\left(u\right)}^{u}\right)}^{t}\le\:1\:\:$$

Furthermore, the RD is well-known by:

$$\:{\pi\:}_{{}^{\circ}F }\left(u\right)={\left(1-\left({\left({m}_{{}^{\circ}F }{\left(u\right)}^{u}\right)}^{t}+{\left({a}_{{}^{\circ}F }{\left(u\right)}^{u}\right)}^{t}+{\left({n}_{{}^{\circ}F }{\left(u\right)}^{u}\right)}^{t}\right)\right)}^{\frac{1}{t}}$$

### Definition 3

^13^ Let ${{}^{\circ}F }_{1}=([{m}_{{{}^{\circ}F }_{1}}{\left(u\right)}^{l},{m}_{{{}^{\circ}F }_{1}}{\left(u\right)}^{u}]$, $[{a}_{{{}^{\circ}F }_{1}}{\left(u\right)}^{l},{a}_{{{}^{\circ}F }_{1}}{\left(u\right)}^{u}],[{n}_{{{}^{\circ}F }_{1}}{\left(u\right)}^{l},{n}_{{{}^{\circ}F }_{1}}{\left(u\right)}^{u}])$, $[{n}_{{{}^{\circ}F }_{1}}{\left(u\right)}^{l},{n}_{{{}^{\circ}F }_{1}}{\left(u\right)}^{u}])$, ${{}^{\circ}F }_{2}=([{m}_{{{}^{\circ}F }_{2}}{\left(u\right)}^{l},{m}_{{{}^{\circ}F }_{2}}{\left(u\right)}^{u}],$, $[{a}_{{{}^{\circ}F }_{2}}{\left(u\right)}^{l},{a}_{{{}^{\circ}F }_{2}}{\left(u\right)}^{u}]$, $[{n}_{{{}^{\circ}F }_{2}}{\left(u\right)}^{l},{n}_{{{}^{\circ}F }_{2}}{\left(u\right)}^{u}])$ be two interval-valued t-spherical fuzzy values (IVTSFVs) and$\:\:\rho\:>0$, $\:\:\rho\:$ be any scalar number, then it satisfies the following operations:

$$\:{{}^{\circ}F }_{1}\oplus\:{{}^{\circ}F }_{2}=\left(\begin{array}{c}\sqrt[t]{\begin{array}{c}{\left([{m}_{{{}^{\circ}F }_{1}}{\left(u\right)}^{l},{m}_{{{}^{\circ}F }_{1}}{\left(u\right)}^{u}]\right)}^{t}+{\left([{m}_{{{}^{\circ}F }_{2}}{\left(u\right)}^{l},{m}_{{{}^{\circ}F }_{2}}{\left(u\right)}^{u}]\right)}^{t}\\\:-{\left([{m}_{{{}^{\circ}F }_{1}}{\left(u\right)}^{l},{m}_{{{}^{\circ}F }_{1}}{\left(u\right)}^{u}]\right)}^{t}.{\left([{m}_{{{}^{\circ}F }_{2}}{\left(u\right)}^{l},{m}_{{{}^{\circ}F }_{2}}{\left(u\right)}^{u}]\right)}^{t}\end{array}},\:\\\:\left([{a}_{{{}^{\circ}F }_{1}}{\left(u\right)}^{l},{a}_{{{}^{\circ}F }_{1}}{\left(u\right)}^{u}].[{a}_{{{}^{\circ}F }_{2}}{\left(u\right)}^{l},{a}_{{{}^{\circ}F }_{2}}{\left(u\right)}^{u}]\right),\left([{n}_{{{}^{\circ}F }_{1}}{\left(u\right)}^{l},{n}_{{{}^{\circ}F }_{1}}{\left(u\right)}^{u}].[{n}_{{{}^{\circ}F }_{2}}{\left(u\right)}^{l},{n}_{{{}^{\circ}F }_{2}}{\left(u\right)}^{u}]\right)\end{array}\right)$$

$$\:{{}^{\circ}F }_{1}\otimes\:{{}^{\circ}F }_{2}=\left(\begin{array}{c}\left([{m}_{{{}^{\circ}F }_{1}}{\left(u\right)}^{l},{m}_{{{}^{\circ}F }_{1}}{\left(u\right)}^{u}].[{m}_{{{}^{\circ}F }_{2}}{\left(u\right)}^{l},{m}_{{{}^{\circ}F }_{2}}{\left(u\right)}^{u}]\right),\sqrt[t]{\begin{array}{c}{\left([{a}_{{{}^{\circ}F }_{1}}{\left(u\right)}^{l},{a}_{{{}^{\circ}F }_{1}}{\left(u\right)}^{u}]\right)}^{t}+{\left([{a}_{{{}^{\circ}F }_{2}}{\left(u\right)}^{l},{a}_{{{}^{\circ}F }_{2}}{\left(u\right)}^{u}]\right)}^{t}\\\:-{\left([{a}_{{{}^{\circ}F }_{1}}{\left(u\right)}^{l},{a}_{{{}^{\circ}F }_{1}}{\left(u\right)}^{u}]\right)}^{t}.{\left([{a}_{{{}^{\circ}F }_{2}}{\left(u\right)}^{l},{a}_{{{}^{\circ}F }_{2}}{\left(u\right)}^{u}]\right)}^{t}\end{array}},\\\:\sqrt[t]{\begin{array}{c}{\left([{n}_{{{}^{\circ}F }_{1}}{\left(u\right)}^{l},{n}_{{{}^{\circ}F }_{1}}{\left(u\right)}^{u}]\right)}^{t}+{\left([{n}_{{{}^{\circ}F }_{2}}{\left(u\right)}^{l},{n}_{{{}^{\circ}F }_{2}}{\left(u\right)}^{u}]\right)}^{t}\\\:-{\left([{n}_{{{}^{\circ}F }_{1}}{\left(u\right)}^{l},{n}_{{{}^{\circ}F }_{1}}{\left(u\right)}^{u}]\right)}^{t}.{\left([{n}_{{{}^{\circ}F }_{2}}{\left(u\right)}^{l},{n}_{{{}^{\circ}F }_{2}}{\left(u\right)}^{u}]\right)}^{t}\end{array}}\end{array}\right)$$

$$\:{\uprho\:}.{{}^{\circ}F }_{1}=\left(\sqrt[t]{1-{\left(1-{\left([{m}_{{{}^{\circ}F }_{1}}{\left(u\right)}^{l},{m}_{{{}^{\circ}F }_{1}}{\left(u\right)}^{u}]\right)}^{t}\right)}^{\rho\:}},{\left([{a}_{{{}^{\circ}F }_{1}}{\left(u\right)}^{l},{a}_{{{}^{\circ}F }_{1}}{\left(u\right)}^{u}]\right)}^{{\uprho\:}},{\left([{n}_{{{}^{\circ}F }_{1}}{\left(u\right)}^{l},{n}_{{{}^{\circ}F }_{1}}{\left(u\right)}^{u}]\right)}^{{\uprho\:}}\right)$$

$$\:\:{{}^{\circ}F }_{1}^{\rho\:}=\left({\left([{m}_{{{}^{\circ}F }_{1}}{\left(u\right)}^{l},{m}_{{{}^{\circ}F }_{1}}{\left(u\right)}^{u}]\right)}^{{\uprho\:}},\sqrt[t]{1-{\left(1-{\left([{a}_{{{}^{\circ}F }_{1}}{\left(u\right)}^{l},{a}_{{{}^{\circ}F }_{1}}{\left(u\right)}^{u}]\right)}^{\text{t}}\right)}^{{\uprho\:}}},\sqrt[t]{1-{\left(1-{\left([{n}_{{{}^{\circ}F }_{1}}{\left(u\right)}^{l},{n}_{{{}^{\circ}F }_{1}}{\left(u\right)}^{u}]\right)}^{\text{t}}\right)}^{{\uprho\:}}}\:\right)$$

$$\:{{}^{\circ}F }_{1}^{c}=\left([{n}_{{{}^{\circ}F }_{1}}{\left(u\right)}^{l},{n}_{{{}^{\circ}F }_{1}}{\left(u\right)}^{u}],\:[{a}_{{{}^{\circ}F }_{1}}{\left(u\right)}^{l},{a}_{{{}^{\circ}F }_{1}}{\left(u\right)}^{u}],[{m}_{{{}^{\circ}F }_{1}}{\left(u\right)}^{l},{m}_{{{}^{\circ}F }_{1}}{\left(u\right)}^{u}]\right)$$

Additionally, the following steps can be used to add and multiply IVTSFSs.

$$\:{{}^{\circ}F }_{1}\oplus\:{{}^{\circ}F }_{2}={{}^{\circ}F }_{2}\oplus\:{{}^{\circ}F }_{1}$$

$$\:{{}^{\circ}F }_{1}\otimes\:{{}^{\circ}F }_{2}={{}^{\circ}F }_{2}\otimes\:{{}^{\circ}F }_{1}$$

### Definition 4

The score and accuracy functions have been created to evaluate and compare IVTSFS. Equations (3) and (4) provide the accuracy and score functions for each IVTSFS.

Score function:

3 $$\:{Sc}_{{}^{\circ}F }=\frac{{\left(\left[{m}_{{}^{\circ}F }^{l}+{m}_{{}^{\circ}F }^{u}\right]-\left[{n}_{{}^{\circ}F }^{l}+{n}_{{}^{\circ}F }^{u}\right]\:\right)}^{t}-{\left(\left[{a}_{{}^{\circ}F }^{l}+{a}_{{}^{\circ}F }^{u}\right]-\left[\left({n}_{{}^{\circ}F }^{l}+{n}_{{}^{\circ}F }^{u}\right)\:\right]\right)}^{t}\:\:\:}{2}$$

Accuracy function:

4 $$\:{Ac}_{{}^{\circ}F }=\frac{\left[{{m}^{l}}_{{}^{\circ}F }^{t}+{{m}^{u}}_{{}^{\circ}F }^{t}\right]+\left[{{a}^{l}}_{{}^{\circ}F }^{t}+{{a}^{u}}_{{}^{\circ}F }^{t}\right]+\left[{{n}^{l}}_{{}^{\circ}F }^{t}+{{n}^{u}}_{{}^{\circ}F }^{t}\right]}{2}$$

## IVTSF-CoCoSo algorithm

**Fig. 1:**
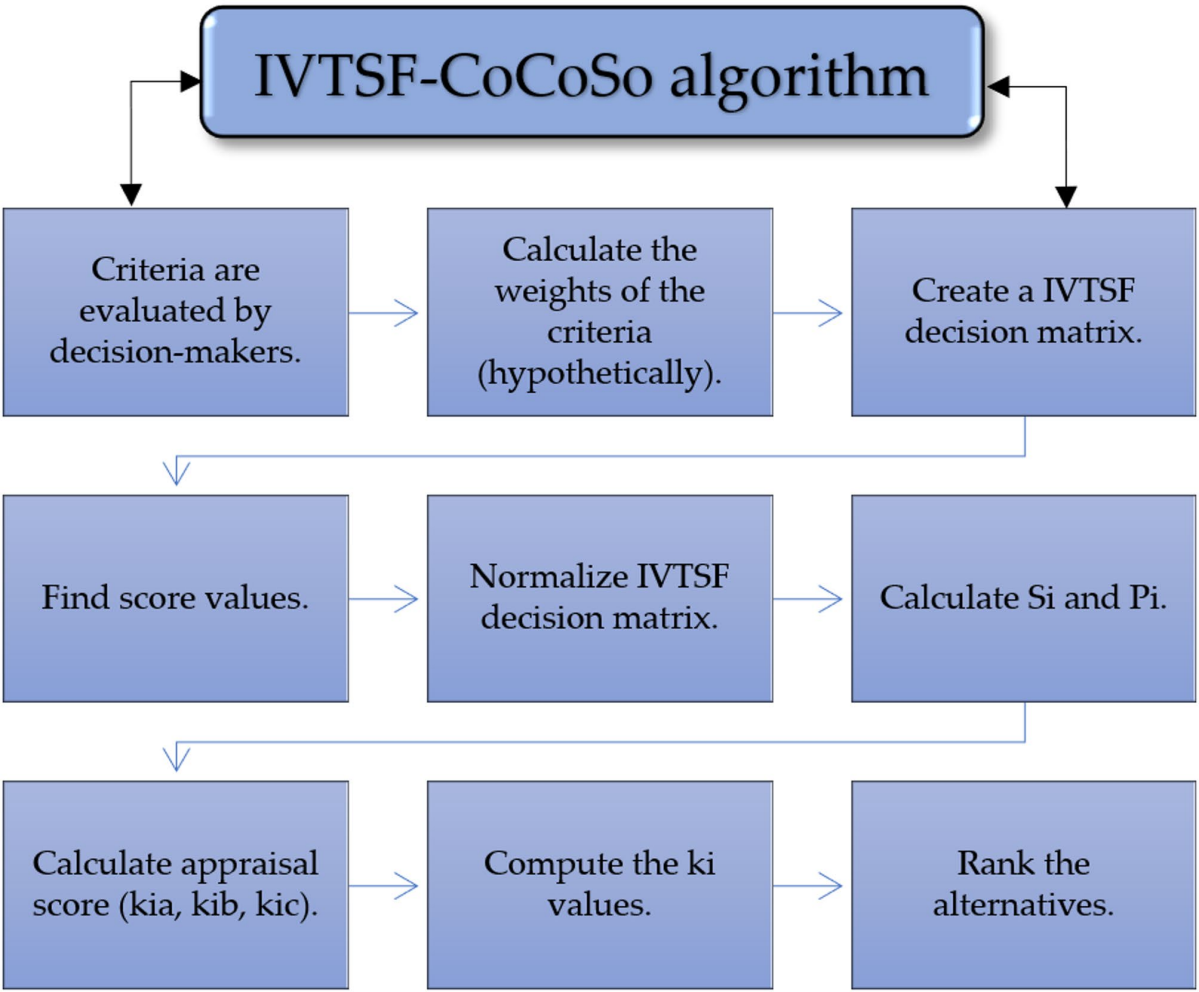
Flowchart of the methodology.

IVTSF-CoCoSo represents a combination of IVTSFS with CoCoSo for creating better decisions when operating within uncertain conditions. This method’s evaluation process enables flexible, accurate, and robust alternative assessment that works optimally for complex choices such as martial arts referee selection. The choice of IVTSFS is inspired by the desire to represent complex and uncertain linguistic judgments more expressively and realistically. Even in the case of referee selection in martial arts, where the experts are involved, there are always some hesitations, subjectivity, and vagueness in their evaluations. The combination of the strengths enables IVTSFS to have experts describe assessments in terms of interval values and consider the hesitancy and multi-dimensional uncertainty. CoCoSo method, however, incorporates multiple ranking principles, including the weighted sum model, multiplicative model, and relative importance aggregation, which makes it a powerful CoCoSo method MCDM tool. Its compromise solutions capability considers both the performance of individual criteria and the international significance, and makes it applicable in referee evaluation problems. By combining IVTSFS with CoCoSo, the study has provided a realistic and complete model to deal with imprecise expert input and produce credible referee rankings. The following are the detailed steps of IVTSF-CoCoSo MCDM algorithm as shown in Fig. 1.

### Step 1

Development of the initial decision matrix.

The initial stage in MCDM approaches is to create a decision matrix, as shown below. Consider $\:R=\left\{{R}_{1},{R}_{2},\cdots\:,{R}_{m}\right\}$ as the collection of alternatives, $\:C=\left\{{C}_{1},{C}_{2},\cdots\:,{C}_{j},\cdots\:,{C}_{n}\right\}$ representing the specified criteria, and $\:W=\left\{{w}_{1},{w}_{2},\cdots\:,{w}_{n}\right\}$ as the array of weights with $\:{w}_{j}\in\:\left[\text{0,1}\right]$. The matrix $\:{}^{\circ}F ={\left({{}^{\circ}F }_{ij}\right)}_{m\times\:n}$ represents the evaluation of choice A by the decision-maker number $\:D$ using criterion $\:n$. The matrix is generated using linguistic concepts.

5 $$\:{}^{\circ}F ={\left({C}_{j}\left({d}_{i}\right)\right)}_{m\times\:n}=\left[\begin{array}{ccc}{{}^{\circ}F }_{11}&\:\cdots\:&\:{{}^{\circ}F }_{1n}\\\: \vdots &\:\ddots\:&\: \vdots \\\:{{}^{\circ}F }_{m1}&\:\cdots\:&\:{{}^{\circ}F }_{mn}\end{array}\right]$$

### Step 2

Transforming linguistic variables into IVTSFVs.

In the second stage, the identified linguistic variables from step 1 are transformed to IVTSFVs using Table 1, and the decision matrix is generated using Eq. (6).

6 $$\:{}^{\circ}F ={\left({C}_{j}\left({d}_{i}\right)\right)}_{m\times\:n}=\left[\begin{array}{ccc}{f}_{11}&\:\cdots\:&\:{f}_{1\text{n}}\\\: \vdots &\:\ddots\:&\: \vdots \\\:{f}_{\text{m}1}&\:\cdots\:&\:{f}_{\text{m}\text{n}}\end{array}\right]$$

### Step 3

The creation of an Aggregated Decision Matrix.

This stage involves combining experts’ opinions while considering the specified weight for each opinion. The aggregated decision matrix was then created using the IVTSFWA operators, as shown in Eq. (7).

7 $$\:IVTSFWA=\left\{\begin{array}{c}\left[{\left(1-{\prod\:}_{\mathfrak{i}=1}^{n}{\left(1-{{m}^{r}}^{t}\right)}^{{w}_{\mathfrak{i}}}\right)}^{\raisebox{1ex}{$1$}\!\left/\:\!\raisebox{-1ex}{$t$}\right.},{\left(1-{\prod\:}_{\mathfrak{i}=1}^{n}{\left(1-{{m}^{i}}^{t}\right)}^{{w}_{\mathfrak{i}}}\right)}^{\raisebox{1ex}{$1$}\!\left/\:\!\raisebox{-1ex}{$t$}\right.}\right],\\\:\left[{\prod\:}_{\mathfrak{i}=1}^{n}{{n}^{r}}^{{w}_{\mathfrak{i}}},{\prod\:}_{\mathfrak{i}=1}^{n}{{n}^{i}}^{{w}_{\mathfrak{i}}}\right],\\\:\left[\begin{array}{c}{\left({\prod\:}_{\mathfrak{i}=1}^{n}{\left(1-{{m}^{r}}^{t}\right)}^{{w}_{\mathfrak{i}}}-{\prod\:}_{\mathfrak{i}=1}^{n}{\left(1-{{m}^{r}}^{t}-{{a}^{r}}^{t}\right)}^{{w}_{\mathfrak{i}}}\right)}^{\raisebox{1ex}{$1$}\!\left/\:\!\raisebox{-1ex}{$t$}\right.},\\\:{\left({\prod\:}_{\mathfrak{i}=1}^{n}{\left(1-{{m}^{i}}^{t}\right)}^{{w}_{\mathfrak{i}}}-{\prod\:}_{\mathfrak{i}=1}^{n}{\left(1-{{m}^{i}}^{t}-{{a}^{i}}^{t}\right)}^{{w}_{\mathfrak{i}}}\right)}^{\raisebox{1ex}{$1$}\!\left/\:\!\raisebox{-1ex}{$t$}\right.}\end{array}\right]\end{array}\right\}\:$$

### Step 4

Computation of the Score function.

Using Eq. 1, the score value for each IVTSFV is calculated, resulting in the matrix $\:{{}^{\circ}F }^{\text{*}}={\left({f}_{ij}^{\text{*}}\right)}_{m\times\:n}$.

### Step 5

The decision matrix’s normalization.

Normalization is generally used across all MCDM approaches. In this stage, Eq. (8) is used to modify the decision matrix for both positive and negative variables based on the relationships illustrated below. $\:{f}_{j+}^{\text{*}}$ and $\:{f}_{j-}^{\text{*}}$ are the maximum and minimum values for each variable column, respectively.

8 $$\:\frac{{}^{\circ}F }{{{}^{\circ}F }_{ij}}=\left\{\begin{array}{ll}\frac{{f}_{ij}^{\text{*}}-{f}_{{j}^{-}}^{\text{*}}}{{f}_{{j}^{+}}^{\text{*}}-{f}_{{j}^{-}}^{\text{*}}}&\:\text{\:if\:}j\in\:B\\\:\frac{{f}_{{j}^{+}}^{\text{*}}-{f}_{ij}^{\text{*}}}{{f}_{{j}^{+}}^{\text{*}}-{f}_{{j}^{-}}^{\text{*}}}&\:\text{\:if\:}j\in\:C\end{array}\right.\:\:$$

where,

9 $$\:{f}_{{j}^{-}}^{\text{*}}=\underset{i}{\text{m}\text{i}\text{n}}\:{f}_{ij}^{\text{*}}\text{\:and\:}{f}_{{j}^{+}}^{\text{*}}=\underset{i}{\text{m}\text{a}\text{x}}\:{f}_{ij}^{\text{*}}$$

### Step 6

Calculate the power weight of comparability and the sum of weighted comparability sequences.

At this point, the power weight of comparability ($\:{P}_{i}$) and cumulative weighted comparability ($\:{S}_{i}$) sequences for each alternative are computed. The CoCoSo technique uses the weight of variables, represented by $\:W$, as input. The values of $\:{S}_{i}$ and $\:{P}_{i}$ were obtained using the SAW and WASPAS techniques, respectively.

10 $$\:\:{P}_{i}=\sum\:_{j=1}^{n}\:\:{\left({f}_{ij}\right)}^{{W}_{j}}\:$$

11 $$\:{S}_{i}=\sum\:_{j=1}^{n}\:\:{W}_{j}{f}_{ij}$$

### Step 7

Evaluation scores are assessed using three unique procedures.

This section provides the scores of the alternatives determined from three different evaluation procedures, using Formulas (11)–(14). Equation (12) expresses the arithmetic mean of the scores for WSM and WPM, while Eq. (13) expresses the relative scores of WSM and WPM to optimal performance. Equation (14) represents an exact equilibrium of WSM and WPM. In Eq. (14), an expert assigns $\:\lambda\:$, however a value of $\:\lambda\:=0.9$ provides greater flexibility.

12 $${k_{ia}}~ = \frac{{{P_i} + {S_i}}}{{\mathop \sum \nolimits_{i = 1}^m \left( {{P_i} + {S_i}} \right)}}$$

13 $${k_{ib}} = \frac{{{S_i}}}{{\mathop {{\text{min}}}\limits_i {S_i}}} + \frac{{{P_i}}}{{\mathop {{\text{min}}}\limits_i {P_i}}}$$

14 $$~{k_{ic}} = \frac{{\lambda {S_i} + \left( {1 - \lambda } \right){P_i}}}{{\lambda \mathop {{\text{max}}}\limits_i {S_i} + \left( {1 - \lambda } \right)\mathop {{\text{max}}}\limits_i {P_i}}}{\text{~}}0 \leqslant \lambda \leqslant 1$$

### Step 8

Evaluation of the final score and ranking of the alternatives.

This section defines the process of calculating the final score using Eq. 15, which illustrates the combination of the arithmetic mean and geometric mean derived from the preceding three strategies. Consequently, the alternatives with the highest$\:\:{k}_{i}$ score are the most optimal.

15 $$\:{k}_{i}={\left({k}_{ia}*{k}_{ib}{*k}_{ic}\right)}^{\frac{1}{3}}+\frac{1}{3}\left({k}_{ia}+{k}_{i\text{b}}+{k}_{ic}\right)$$

## Assessment of referee selection in martial arts

Martial arts encompass various combat sports and self-defense techniques, emphasizing discipline, method, and strategy. The popular martial arts disciplines are karate, judo, taekwondo, and Muay Thai with their own set of game rules and unique point-scoring regulations. During competitive martial arts events, referees detect infringements of rules while evaluating techniques, which results in live decisions that determine contest results. Referees work under intense pressure, yet must preserve impartiality; therefore, an organized selection system must find officials who demonstrate top competency.

The selection of martial arts referees hinges on evaluation factors involving professional experience, accurate decision-making, and consistency standards. The MCDM method CoCoSo utilizes expert judgments to develop a ranking system for alternatives. The combined performance measurement methodology identifies referees for important martial arts competitions based on their qualifications. The CoCoSo methodology enables organizations to create ranked referee assessments through examinations of both quantitative and qualitative evaluation characteristics.

Real-life decision-making must deal with subjective evaluations because experienced referees differ in their expertise levels with each other. IVTSFS provides decision-makers with enhanced flexibility for expressing their preferences as a solution to this problem. IVTSFS enables users to express doubt and doubtfulness, resulting in more exact measurements. Our selection framework unites CoCoSo with IVTSFS to guarantee the most appropriate referee selection by relying on complete assessment standards.

The study includes analysis of ten potential referee candidates ($\:{R}_{1},{R}_{2},{R}_{3},{R}_{4},{R}_{5},{R}_{6},{R}_{7},{R}_{8},{R}_{9},\:{R}_{10}$). The ten referees considered in this research were pre-screened applicants taken from the referee pool of the national martial arts federation. They had at least two regional or national events in the past two years. The expert panel (head of officiating committee, senior referee trainer, and international judge) had previously worked with or viewed each candidate in practice. Such experts were therefore better placed to rate the performance of the referees based on the seven criteria on linguistic ratings of uncertainty, as the IVTSFS framework indicates. In subjective measures like communication skills and stress management, the ranking was done according to the collective view and professional opinion of the experts in the career of the referees. Each expert filled in an evaluation form involving individual responses to minimize influence or bias. The three-expert decision-makers execute the evaluation process.

- $\:{D}_{1}$: Head of Officiating Committee
- $\:{D}_{2}$: Senior Referee Trainer
- $$\:{D}_{3}$$: International Judge

The seven critical criteria guide each decision-maker in evaluating referees. These criteria are vital in choosing suitable referees during the selection process.

- Experience ($\:{C}_{1}$): A referee’s expertise represents the core requirement for their experience based on their work officiating martial arts events at national and international levels. A referee builds up experience through extensive service, enhancing their ability to manage stressful situations while mastering gameplay tactics and comprehending match situations correctly. The evaluation of experience depends on the length of time spent officiating, the management of various competition levels, and the different martial arts disciplines refereed.
- Decision Accuracy ($\:{C}_{2}$): The main factor preserving competition fairness depends on referees providing consistent and mitigating biased judgments. Multiple factors come into play to evaluate decision accuracy, which unite past performance analysis results, judgment error data, and consistency evaluation of matches. Sports integrity stays intact, and controversies decrease when decision-makers select accurate choices.
- Rule Knowledge ($\:{C}_{3}$): Comprehending every rule and regulation in martial arts is a fundamental requirement for refereeing. A test requires referees to display full knowledge of every competition procedural guideline, starting with scoring protocols and extending to disciplinary mandates. The referees’ thorough knowledge of rules permits them to evaluate their meaning quickly, leading to proper enforcement to stop unfair situations and disputes.
- Physical Fitness ($\:{C}_{4}$): Referee-related physical excellence enables correct movements, quick movement tracking, and prolonged concentration focus across tournament events. The passing of this requirement proves that referees can move quickly and have good stamina for enduring whole matches before execution. They maintain high situational awareness when speed becomes important for matches.
- Stress Management ($\:{C}_{5}$): Competing to officiate martial arts competitions requires complete composure even during intense match situations, often leading to resistance from both athletes and coaches. Referee dedication to mental strength and personal understanding brings professional competence to dispute settlement at work. Official stress management competence leads to consistent fair and stable match decisions.
- Consistency ($\:{C}_{6}$): Referees need to enforce decisions similarly throughout various matches and tournaments. The unpredictable nature of officiating causes contestants to question the fairness of martial arts competitions, harming their perception of such events. Steady application of rules and scoring methods without personal favoritism forms the essence of this evaluation standard for referees to demonstrate consistent decision-making.
- Communication Skills ($\:{C}_{7}$): Efficient communication is crucial in referees’ judgments to athletes, coaches, and other officiating staff. Referee proficiency depends on their capacity to express their calls with penalty explanations while using professional language to reduce conflict. Excellent communication abilities help officials create smooth matches, leading to fair acceptance of their decisions.

The criteria used in the present research have been chosen using a literature review and consulting experts. Earlier research in sports officiating highlights experience and knowledge of the rules as the primary competencies that referees should possess. In the meantime, the decisions’ accuracy, consistency, and communication ability are often noted as performance-related traits contributing to in-game performance and justice. In addition, the importance of physical fitness and stress management cannot be underestimated in the martial arts because the matches are speedy and pressured. To ascertain the suitability of these criteria, three domain experts, the head of the officiating committee, a senior referee trainer, and an international judge, confirmed the necessity and comprehensiveness of these criteria in determining the suitability of martial arts referees.

Several realistic constraints bound the referee selection problem that is the subject of this study. Ten referee candidates ($\:{R}_{1}$ to $\:{R}_{10}$) were considered, and all of them had passed the minimum eligibility requirements by the national martial arts officiating organization. In such a tournament, 3–5 referees are chosen, based on the competition format, size, and program. Although the main output of this study is a ranking of the candidates to select the most suitable candidates, the final selection could be scaled to various selection quotas by choosing the best referees based on the final ranking. Moreover, experts established seven key criteria to evaluate each referee, and the assessment was collected among three decision-makers, who represent the different and authoritative levels in the officiating structure. Such limitations reflect those in the real world and make the model developed viable and implementable.

The CoCoSo MCDM algorithm integrated into the IVTSFS allows us to develop an expert-based system that picks referees from available candidates. The method guarantees equal treatment and transparent detection of reliable officials, resulting in higher officiating performance across martial arts competitions. Table 1 displays the linguistic evaluation of IVTSFS.

**Table 1:** Linguistic evaluation of interval-valued t-spherical fuzzy sets.

Linguistic terms	Interval-valued t-spherical fuzzy values					
	$\:{m}^{l}$	$\:{m}^{u}$	$\:{n}^{l}$	$\:{n}^{u}$	$\:{a}^{l}$	$\:{a}^{u}$
Absolutely more important (AMI)	$\:0.85$	$\:0.95$	$\:0.25$	$\:0.30$	$\:0.20$	$\:0.25$
Very high important (VHI)	$\:0.75$	$\:0.85$	$\:0.30$	$\:0.35$	$\:0.25$	$\:0.30$
Highly important (HI)	$\:0.65$	$\:0.75$	$\:0.35$	$\:0.40$	$\:0.30$	$\:0.35$
Slightly more important (SMI)	$\:0.55$	$\:0.65$	$\:0.40$	$\:0.45$	$\:0.35$	$\:0.40$
Equally important (EI)	$\:0.45$	$\:0.55$	$\:0.45$	$\:0.50$	$\:0.40$	$\:0.45$
Slightly low important (SLI)	$\:0.35$	$\:0.45$	$\:0.50$	$\:0.55$	$\:0.45$	$\:0.50$
Low important (LI)	$\:0.25$	$\:0.35$	$\:0.55$	$\:0.60$	$\:0.50$	$\:0.55$
Very low important (VLI)	$\:0.15$	$\:0.25$	$\:0.60$	$\:0.65$	$\:0.55$	$\:0.60$
Absolutely low important (ALI)	$\:0.05$	$\:0.15$	$\:0.65$	$\:0.70$	$\:0.60$	$\:0.65$

Table 2 is the IVTSF decision matrix, which is based on linguistic evaluations in Table 1. The seven criteria ($\:\text{C}_{1}$ to $\:{\text{C}}_{7}$) are applied to each referee ($\:{\text{R}}_{1}$ to $\:{\text{R}}_{10}$) by three decision-makers ($\:{\text{D}}_{1}$ to$\:\:{\text{D}}_{3}$) who are independent of one another. Linguistic terms employed in the expert knowledge are identified with matching IVTSFVs. Based on these values, the IVTSF decision matrix is constructed to be further processed in the CoCoSo algorithm. In this structure, the individual expert assessments are kept separate to have multi-expert evaluation variability.

**Table 2:** Interval-valued t-spherical fuzzy decision matrix.

	$\:{\varvec{C}}_{1}$			$\:{\varvec{C}}_{2}$			$\:{\varvec{C}}_{3}$			$\:{\varvec{C}}_{4}$			$\:{\varvec{C}}_{5}$			$\:{\varvec{C}}_{6}$			$\:{\varvec{C}}_{7}$		
	$\:{D}_{1}$	$\:{D}_{2}$	$\:{D}_{3}$	$\:{D}_{1}$	$\:{D}_{2}$	$\:{D}_{3}$	$\:{D}_{1}$	$\:{D}_{2}$	$\:{D}_{3}$	$\:{D}_{1}$	$\:{D}_{2}$	$\:{D}_{3}$	$\:{D}_{1}$	$\:{D}_{2}$	$\:{D}_{3}$	$\:{D}_{1}$	$\:{D}_{2}$	$\:{D}_{3}$	$\:{D}_{1}$	$\:{D}_{2}$	$\:{D}_{3}$
$\:{\varvec{R}}_{1}$	$\:HI$	$\:SMI$	$\:EI$	$\:SMI$	$\:VHI$	$\:SMI$	$\:HI$	$\:VHI$	$\:HI$	$\:HI$	$\:SMI$	$\:EI$	$\:SMI$	$\:EI$	$\:EI$	$\:SMI$	$\:HI$	$\:SMI$	$\:HI$	$\:SMI$	$\:EI$
$\:{\varvec{R}}_{2}$	$\:SMI$	$\:VHI$	$\:EI$	$\:VHI$	$\:VHI$	$\:HI$	$\:SMI$	$\:HI$	$\:EI$	$\:SMI$	$\:HI$	$\:EI$	$\:VHI$	$\:VHI$	$\:SMI$	$\:VHI$	$\:EI$	$\:HI$	$\:EI$	$\:HI$	$\:VHI$
$\:{\varvec{R}}_{3}$	$\:HI$	$\:SMI$	$\:VHI$	$\:SMI$	$\:HI$	$\:SMI$	$\:VHI$	$\:HI$	$\:VHI$	$\:HI$	$\:HI$	$\:VHI$	$\:SMI$	$\:HI$	$\:HI$	$\:SMI$	$\:VHI$	$\:SMI$	$\:VHI$	$\:HI$	$\:HI$
$\:{\varvec{R}}_{4}$	$\:AMI$	$\:HI$	$\:VHI$	$\:AMI$	$\:HI$	$\:HI$	$\:SMI$	$\:VHI$	$\:HI$	$\:HI$	$\:VHI$	$\:VHI$	$\:HI$	$\:HI$	$\:HI$	$\:AMI$	$\:HI$	$\:HI$	$\:HI$	$\:VHI$	$\:HI$
$\:{\varvec{R}}_{5}$	$\:VHI$	$\:HI$	$\:HI$	$\:VHI$	$\:VHI$	$\:SMI$	$\:SLI$	$\:EI$	$\:SMI$	$\:VHI$	$\:EI$	$\:HI$	$\:HI$	$\:VHI$	$\:VHI$	$\:VHI$	$\:SMI$	$\:SMI$	$\:SMI$	$\:EI$	$\:VHI$
$\:{\varvec{R}}_{6}$	$\:HI$	$\:HI$	$\:HI$	$\:HI$	$\:HI$	$\:VHI$	$\:HI$	$\:VHI$	$\:HI$	$\:VHI$	$\:HI$	$\:VHI$	$\:VHI$	$\:HI$	$\:HI$	$\:HI$	$\:HI$	$\:VHI$	$\:HI$	$\:HI$	$\:HI$
$\:{\varvec{R}}_{7}$	$\:SMI$	$\:HI$	$\:VHI$	$\:SMI$	$\:SMI$	$\:SMI$	$\:VHI$	$\:VHI$	$\:HI$	$\:HI$	$\:HI$	$\:VHI$	$\:HI$	$\:SMI$	$\:VHI$	$\:SMI$	$\:HI$	$\:SMI$	$\:HI$	$\:HI$	$\:SMI$
$\:{\varvec{R}}_{8}$	$\:EI$	$\:HI$	$\:SMI$	$\:EI$	$\:HI$	$\:VHI$	$\:VHI$	$\:EI$	$\:HI$	$\:HI$	$\:EI$	$\:SMI$	$\:HI$	$\:HI$	$\:HI$	$\:EI$	$\:HI$	$\:VHI$	$\:HI$	$\:EI$	$\:HI$
$\:{\varvec{R}}_{9}$	$\:SLI$	$\:SMI$	$\:EI$	$\:SLI$	$\:HI$	$\:VHI$	$\:EI$	$\:HI$	$\:VHI$	$\:HI$	$\:HI$	$\:EI$	$\:SMI$	$\:HI$	$\:HI$	$\:SLI$	$\:VHI$	$\:VHI$	$\:VHI$	$\:HI$	$\:HI$
$\:{\varvec{R}}_{10}$	$\:LI$	$\:VHI$	$\:HI$	$\:LI$	$\:SMI$	$\:VHI$	$\:SMI$	$\:EI$	$\:HI$	$\:HI$	$\:EI$	$\:HI$	$\:VHI$	$\:SMI$	$\:HI$	$\:LI$	$\:HI$	$\:VHI$	$\:HI$	$\:EI$	$\:SMI$

By using Eq. (5), aggregate the IVTSFVs as shown in Table 3. Weights of the criteria are$\:\:{\left(\text{0.07,0.13,0.01,0.23,0.30,0.09,0.17}\right)}^{T}$, and these weights are taken hypothetically.

**Table 3:** Interval-valued t-spherical fuzzy aggregated decision matrix.

	$\:{\varvec{C}}_{1}$						$\:{\varvec{C}}_{2}$						$\:{\varvec{C}}_{3}$					
	$\:{m}^{l}$	$\:{m}^{u}$	$\:{n}^{l}$	$\:{n}^{u}$	$\:{a}^{l}$	$\:{a}^{u}$	$\:{m}^{l}$	$\:{m}^{u}$	$\:{n}^{l}$	$\:{n}^{u}$	$\:{a}^{l}$	$\:{a}^{u}$	$\:{m}^{l}$	$\:{m}^{u}$	$\:{n}^{l}$	$\:{n}^{u}$	$\:{a}^{l}$	$\:{a}^{u}$
$\:{\varvec{R}}_{1}$	$\:0.39$	$\:0.46$	$\:0.82$	$\:0.84$	$\:0.25$	$\:0.29$	$\:0.52$	$\:0.61$	$\:0.67$	$\:0.71$	$\:0.26$	$\:0.31$	$\:0.30$	$\:0.35$	$\:0.97$	$\:0.97$	$\:0.13$	$\:0.16$
$\:{\varvec{R}}_{2}$	$\:0.43$	$\:0.51$	$\:0.82$	$\:0.84$	$\:0.24$	$\:0.28$	$\:0.58$	$\:0.68$	$\:0.64$	$\:0.68$	$\:0.22$	$\:0.28$	$\:0.24$	$\:0.29$	$\:0.97$	$\:0.98$	$\:0.15$	$\:0.18$
$\:{\varvec{R}}_{3}$	$\:0.46$	$\:0.54$	$\:0.80$	$\:0.82$	$\:0.22$	$\:0.26$	$\:0.47$	$\:0.56$	$\:0.69$	$\:0.72$	$\:0.27$	$\:0.32$	$\:0.31$	$\:0.37$	$\:0.97$	$\:0.97$	$\:0.12$	$\:0.15$
$\:{\varvec{R}}_{4}$	$\:0.54$	$\:0.65$	$\:0.78$	$\:0.80$	$\:0.19$	$\:0.24$	$\:0.61$	$\:0.72$	$\:0.64$	$\:0.67$	$\:0.23$	$\:0.28$	$\:0.29$	$\:0.34$	$\:0.97$	$\:0.97$	$\:0.13$	$\:0.16$
$\:{\varvec{R}}_{5}$	$\:0.48$	$\:0.56$	$\:0.79$	$\:0.82$	$\:0.20$	$\:0.25$	$\:0.57$	$\:0.66$	$\:0.65$	$\:0.69$	$\:0.24$	$\:0.29$	$\:0.20$	$\:0.24$	$\:0.98$	$\:0.98$	$\:0.17$	$\:0.20$
$\:{\varvec{R}}_{6}$	$\:0.45$	$\:0.53$	$\:0.80$	$\:0.82$	$\:0.21$	$\:0.26$	$\:0.56$	$\:0.65$	$\:0.65$	$\:0.69$	$\:0.23$	$\:0.28$	$\:0.30$	$\:0.35$	$\:0.97$	$\:0.97$	$\:0.13$	$\:0.16$
$\:{\varvec{R}}_{7}$	$\:0.46$	$\:0.54$	$\:0.80$	$\:0.82$	$\:0.22$	$\:0.26$	$\:0.44$	$\:0.52$	$\:0.70$	$\:0.73$	$\:0.28$	$\:0.33$	$\:0.31$	$\:0.37$	$\:0.97$	$\:0.97$	$\:0.12$	$\:0.15$
$\:{\varvec{R}}_{8}$	$\:0.39$	$\:0.46$	$\:0.82$	$\:0.84$	$\:0.25$	$\:0.29$	$\:0.53$	$\:0.62$	$\:0.67$	$\:0.71$	$\:0.27$	$\:0.31$	$\:0.28$	$\:0.33$	$\:0.97$	$\:0.97$	$\:0.14$	$\:0.17$
$\:{\varvec{R}}_{9}$	$\:0.32$	$\:0.39$	$\:0.84$	$\:0.86$	$\:0.28$	$\:0.32$	$\:0.52$	$\:0.61$	$\:0.68$	$\:0.72$	$\:0.29$	$\:0.33$	$\:0.28$	$\:0.33$	$\:0.97$	$\:0.97$	$\:0.14$	$\:0.17$
$\:{\varvec{R}}_{10}$	$\:0.45$	$\:0.53$	$\:0.82$	$\:0.84$	$\:0.27$	$\:0.31$	$\:0.50$	$\:0.58$	$\:0.70$	$\:0.74$	$\:0.32$	$\:0.36$	$\:0.24$	$\:0.29$	$\:0.97$	$\:0.98$	$\:0.15$	$\:0.18$
max	$\:0.54$	$\:0.65$	$\:0.84$	$\:0.86$	$\:0.28$	$\:0.32$	$\:0.61$	$\:0.72$	$\:0.70$	$\:0.74$	$\:0.32$	$\:0.36$	$\:0.31$	$\:0.37$	$\:0.98$	$\:0.98$	$\:0.17$	$\:0.20$
min	$\:0.32$	$\:0.39$	$\:0.78$	$\:0.80$	$\:0.19$	$\:0.24$	$\:0.44$	$\:0.52$	$\:0.64$	$\:0.67$	$\:0.22$	$\:0.28$	$\:0.20$	$\:0.24$	$\:0.97$	$\:0.97$	$\:0.12$	$\:0.15$
	$\:{C}_{4}$						$\:{C}_{5}$						$\:{C}_{6}$					
	$\:{m}^{l}$	$\:{m}^{u}$	$\:{n}^{l}$	$\:{n}^{u}$	$\:{a}^{l}$	$\:{a}^{u}$	$\:{m}^{l}$	$\:{m}^{u}$	$\:{n}^{l}$	$\:{n}^{u}$	$\:{a}^{l}$	$\:{a}^{u}$	$\:{m}^{l}$	$\:{m}^{u}$	$\:{n}^{l}$	$\:{n}^{u}$	$\:{a}^{l}$	$\:{a}^{u}$
$\:{\varvec{R}}_{1}$	$\:0.52$	$\:0.62$	$\:0.53$	$\:0.57$	$\:0.33$	$\:0.37$	$\:0.48$	$\:0.58$	$\:0.47$	$\:0.52$	$\:0.38$	$\:0.42$	$\:0.43$	$\:0.51$	$\:0.77$	$\:0.80$	$\:0.25$	$\:0.29$
$\:{\varvec{R}}_{2}$	$\:0.52$	$\:0.62$	$\:0.53$	$\:0.57$	$\:0.33$	$\:0.37$	$\:0.69$	$\:0.79$	$\:0.37$	$\:0.42$	$\:0.28$	$\:0.33$	$\:0.48$	$\:0.57$	$\:0.76$	$\:0.79$	$\:0.25$	$\:0.29$
$\:{\varvec{R}}_{3}$	$\:0.64$	$\:0.73$	$\:0.47$	$\:0.52$	$\:0.26$	$\:0.32$	$\:0.61$	$\:0.71$	$\:0.40$	$\:0.45$	$\:0.31$	$\:0.36$	$\:0.47$	$\:0.56$	$\:0.76$	$\:0.79$	$\:0.24$	$\:0.28$
$\:{\varvec{R}}_{4}$	$\:0.67$	$\:0.77$	$\:0.45$	$\:0.50$	$\:0.25$	$\:0.30$	$\:0.63$	$\:0.73$	$\:0.39$	$\:0.44$	$\:0.29$	$\:0.34$	$\:0.56$	$\:0.67$	$\:0.73$	$\:0.76$	$\:0.21$	$\:0.26$
$\:{\varvec{R}}_{5}$	$\:0.60$	$\:0.70$	$\:0.50$	$\:0.54$	$\:0.30$	$\:0.35$	$\:0.71$	$\:0.81$	$\:0.35$	$\:0.40$	$\:0.26$	$\:0.31$	$\:0.47$	$\:0.56$	$\:0.76$	$\:0.79$	$\:0.24$	$\:0.28$
$\:{\varvec{R}}_{6}$	$\:0.67$	$\:0.77$	$\:0.45$	$\:0.50$	$\:0.25$	$\:0.30$	$\:0.67$	$\:0.78$	$\:0.37$	$\:0.42$	$\:0.28$	$\:0.33$	$\:0.51$	$\:0.60$	$\:0.74$	$\:0.77$	$\:0.22$	$\:0.26$
$\:{\varvec{R}}_{7}$	$\:0.64$	$\:0.73$	$\:0.47$	$\:0.52$	$\:0.26$	$\:0.32$	$\:0.65$	$\:0.76$	$\:0.39$	$\:0.44$	$\:0.30$	$\:0.35$	$\:0.43$	$\:0.51$	$\:0.77$	$\:0.80$	$\:0.25$	$\:0.29$
$\:{\varvec{R}}_{8}$	$\:0.52$	$\:0.62$	$\:0.53$	$\:0.57$	$\:0.33$	$\:0.37$	$\:0.63$	$\:0.73$	$\:0.39$	$\:0.44$	$\:0.29$	$\:0.34$	$\:0.48$	$\:0.57$	$\:0.76$	$\:0.79$	$\:0.25$	$\:0.29$
$\:{\varvec{R}}_{9}$	$\:0.56$	$\:0.65$	$\:0.51$	$\:0.56$	$\:0.31$	$\:0.36$	$\:0.61$	$\:0.71$	$\:0.40$	$\:0.45$	$\:0.31$	$\:0.36$	$\:0.51$	$\:0.60$	$\:0.76$	$\:0.78$	$\:0.26$	$\:0.30$
$\:{\varvec{R}}_{10}$	$\:0.56$	$\:0.65$	$\:0.51$	$\:0.56$	$\:0.31$	$\:0.36$	$\:0.65$	$\:0.76$	$\:0.39$	$\:0.44$	$\:0.30$	$\:0.35$	$\:0.48$	$\:0.56$	$\:0.77$	$\:0.80$	$\:0.29$	$\:0.32$
max	$\:0.67$	$\:0.77$	$\:0.53$	$\:0.57$	$\:0.33$	$\:0.37$	$\:0.71$	$\:0.81$	$\:0.47$	$\:0.52$	$\:0.38$	$\:0.42$	$\:0.56$	$\:0.67$	$\:0.77$	$\:0.80$	$\:0.29$	$\:0.32$
min	$\:0.52$	$\:0.62$	$\:0.45$	$\:0.50$	$\:0.25$	$\:0.30$	$\:0.48$	$\:0.58$	$\:0.35$	$\:0.40$	$\:0.26$	$\:0.31$	$\:0.43$	$\:0.51$	$\:0.73$	$\:0.76$	$\:0.21$	$\:0.26$
	$$\:{C}_{7}$$																	
	$\:{m}^{l}$	$\:{m}^{u}$	$\:{n}^{l}$	$\:{n}^{u}$	$\:{a}^{l}$	$\:{a}^{u}$												
$\:{\varvec{R}}_{1}$	$\:0.48$	$\:0.57$	$\:0.63$	$\:0.66$	$\:0.30$	$\:0.35$												
$\:{\varvec{R}}_{2}$	$\:0.56$	$\:0.66$	$\:0.60$	$\:0.64$	$\:0.28$	$\:0.33$												
$\:{\varvec{R}}_{3}$	$\:0.59$	$\:0.69$	$\:0.57$	$\:0.61$	$\:0.25$	$\:0.30$												
$\:{\varvec{R}}_{4}$	$\:0.59$	$\:0.69$	$\:0.57$	$\:0.61$	$\:0.25$	$\:0.30$												
$\:{\varvec{R}}_{5}$	$\:0.54$	$\:0.63$	$\:0.61$	$\:0.65$	$\:0.30$	$\:0.34$												
$\:{\varvec{R}}_{6}$	$\:0.56$	$\:0.65$	$\:0.59$	$\:0.63$	$\:0.26$	$\:0.31$												
$\:{\varvec{R}}_{7}$	$\:0.53$	$\:0.62$	$\:0.60$	$\:0.64$	$\:0.27$	$\:0.32$												
$\:{\varvec{R}}_{8}$	$\:0.52$	$\:0.61$	$\:0.61$	$\:0.65$	$\:0.29$	$\:0.34$												
$\:{\varvec{R}}_{9}$	$\:0.59$	$\:0.69$	$\:0.57$	$\:0.61$	$\:0.25$	$\:0.30$												
$\:{\varvec{R}}_{10}$	$\:0.48$	$\:0.57$	$\:0.63$	$\:0.66$	$\:0.30$	$\:0.35$												
max	$\:0.59$	$\:0.69$	$\:0.63$	$\:0.66$	$\:0.30$	$\:0.35$												
min	$\:0.48$	$\:0.57$	$\:0.57$	$\:0.61$	$\:0.25$	$\:0.30$												

Use Eq. (1) to find score values as shown in Table 4.

**Table 4:** Score values.

	$\:{\varvec{C}}_{1}$	$\:{\varvec{C}}_{2}$	$\:{\varvec{C}}_{3}$	$\:{\varvec{C}}_{4}$	$\:{\varvec{C}}_{5}$	$\:{\varvec{C}}_{6}$	$\:{\varvec{C}}_{7}$
$\:{\varvec{R}}_{1}$	$\:-0.314$	$\:-0.297$	$\:-0.535$	$\:-0.081$	$\:-0.016$	$\:-0.333$	$\:-0.175$
$\:{\varvec{R}}_{2}$	$\:-0.393$	$\:-0.332$	$\:-0.300$	$\:-0.081$	$\:0.226$	$\:-0.390$	$\:-0.191$
$\:{\varvec{R}}_{3}$	$\:-0.471$	$\:-0.265$	$\:-0.593$	$\:-0.006$	$\:0.086$	$\:-0.391$	$\:-0.197$
$\:{\varvec{R}}_{4}$	$\:-0.585$	$\:-0.322$	$\:-0.486$	$\:0.038$	$\:0.129$	$\:-0.486$	$\:-0.197$
$\:{\varvec{R}}_{5}$	$\:-0.509$	$\:-0.322$	$\:-0.112$	$\:-0.039$	$\:0.269$	$\:-0.391$	$\:-0.190$
$\:{\varvec{R}}_{6}$	$\:-0.460$	$\:-0.327$	$\:-0.535$	$\:0.038$	$\:0.199$	$\:-0.453$	$\:-0.206$
$\:{\varvec{R}}_{7}$	$\:-0.471$	$\:-0.228$	$\:-0.593$	$\:-0.006$	$\:0.156$	$\:-0.333$	$\:-0.201$
$\:{\varvec{R}}_{8}$	$\:-0.314$	$\:-0.293$	$\:-0.441$	$\:-0.081$	$\:0.129$	$\:-0.390$	$\:-0.189$
$\:{\varvec{R}}_{9}$	$\:-0.125$	$\:-0.269$	$\:-0.441$	$\:-0.070$	$\:0.086$	$\:-0.389$	$\:-0.197$
$\:{\varvec{R}}_{10}$	$\:-0.350$	$\:-0.223$	$\:-0.300$	$\:-0.070$	$\:0.156$	$\:-0.317$	$\:-0.175$

We get Table 5 by normalizing the score values using Eqs. (6) and (7). We apply Eqs. (8)–(13) to find the ranking of alternatives shown in Table 6.

**Table 5:** Normalized decision matrix.

	$\:{\varvec{C}}_{1}$	$\:{\varvec{C}}_{2}$	$\:{\varvec{C}}_{3}$	$\:{\varvec{C}}_{4}$	$\:{\varvec{C}}_{5}$	$\:{\varvec{C}}_{6}$	$\:{\varvec{C}}_{7}$
$\:{\varvec{R}}_{1}$	$\:0.589$	$\:0.315$	$\:0.878$	$\:0.000$	$\:0.000$	$\:0.901$	$\:1.000$
$\:{\varvec{R}}_{2}$	$\:0.418$	$\:0.000$	$\:0.390$	$\:0.000$	$\:0.848$	$\:0.565$	$\:0.477$
$\:{\varvec{R}}_{3}$	$\:0.247$	$\:0.611$	$\:1.000$	$\:0.632$	$\:0.359$	$\:0.560$	$\:0.295$
$\:{\varvec{R}}_{4}$	$\:0.000$	$\:0.086$	$\:0.776$	$\:1.000$	$\:0.507$	$\:0.000$	$\:0.295$
$\:{\varvec{R}}_{5}$	$\:0.165$	$\:0.092$	$\:0.000$	$\:0.358$	$\:1.000$	$\:0.560$	$\:0.526$
$\:{\varvec{R}}_{6}$	$\:0.271$	$\:0.042$	$\:0.878$	$\:1.000$	$\:0.755$	$\:0.195$	$\:0.000$
$\:{\varvec{R}}_{7}$	$\:0.247$	$\:0.956$	$\:1.000$	$\:0.632$	$\:0.603$	$\:0.901$	$\:0.145$
$\:{\varvec{R}}_{8}$	$\:0.589$	$\:0.353$	$\:0.684$	$\:0.000$	$\:0.507$	$\:0.565$	$\:0.551$
$\:{\varvec{R}}_{9}$	$\:1.000$	$\:0.581$	$\:0.684$	$\:0.090$	$\:0.359$	$\:0.570$	$\:0.295$
$\:{\varvec{R}}_{10}$	$\:0.511$	$\:1.000$	$\:0.390$	$\:0.090$	$\:0.603$	$\:1.000$	$\:1.000$

**Table 6:** Ranking of alternatives.

	$\:{\varvec{P}}_{\varvec{i}}$	$\:{\varvec{S}}_{\varvec{i}}$	$\:{\varvec{k}}_{\varvec{i}\varvec{a}}$	$\:{\varvec{k}}_{\varvec{i}\varvec{b}}$	$\:{\varvec{k}}_{\varvec{i}\varvec{c}}$	$\:{\varvec{k}}_{\varvec{i}}$	Rank
$\:{\varvec{R}}_{1}$	$\:4.81$	$\:0.34$	$\:0.09$	$\:2.11$	$\:0.65$	$\:1.44$	$\:10$
$\:{\varvec{R}}_{2}$	$\:4.71$	$\:0.42$	$\:0.09$	$\:2.31$	$\:0.70$	$\:1.55$	$\:8$
$\:{\varvec{R}}_{3}$	$\:6.24$	$\:0.46$	$\:0.11$	$\:2.78$	$\:0.86$	$\:1.90$	$\:3$
$\:{\varvec{R}}_{4}$	$\:4.35$	$\:0.45$	$\:0.08$	$\:2.32$	$\:0.70$	$\:1.54$	$\:9$
$\:{\varvec{R}}_{5}$	$\:5.25$	$\:0.55$	$\:0.10$	$\:2.80$	$\:0.84$	$\:1.86$	$\:4$
$\:{\varvec{R}}_{6}$	$\:5.36$	$\:0.51$	$\:0.10$	$\:2.71$	$\:0.82$	$\:1.81$	$\:5$
$\:{\varvec{R}}_{7}$	$\:6.37$	$\:0.58$	$\:0.12$	$\:3.17$	$\:0.96$	$\:2.13$	$\:2$
$\:{\varvec{R}}_{8}$	$\:5.50$	$\:0.39$	$\:0.10$	$\:2.41$	$\:0.75$	$\:1.65$	$\:7$
$\:{\varvec{R}}_{9}$	$\:6.00$	$\:0.38$	$\:0.11$	$\:2.50$	$\:0.78$	$\:1.72$	$\:6$
$\:{\varvec{R}}_{10}$	$\:6.38$	$\:0.63$	$\:0.12$	$\:3.31$	$\:1.00$	$\:2.21$	$\:1$

### Result discussion

**Fig. 2:**
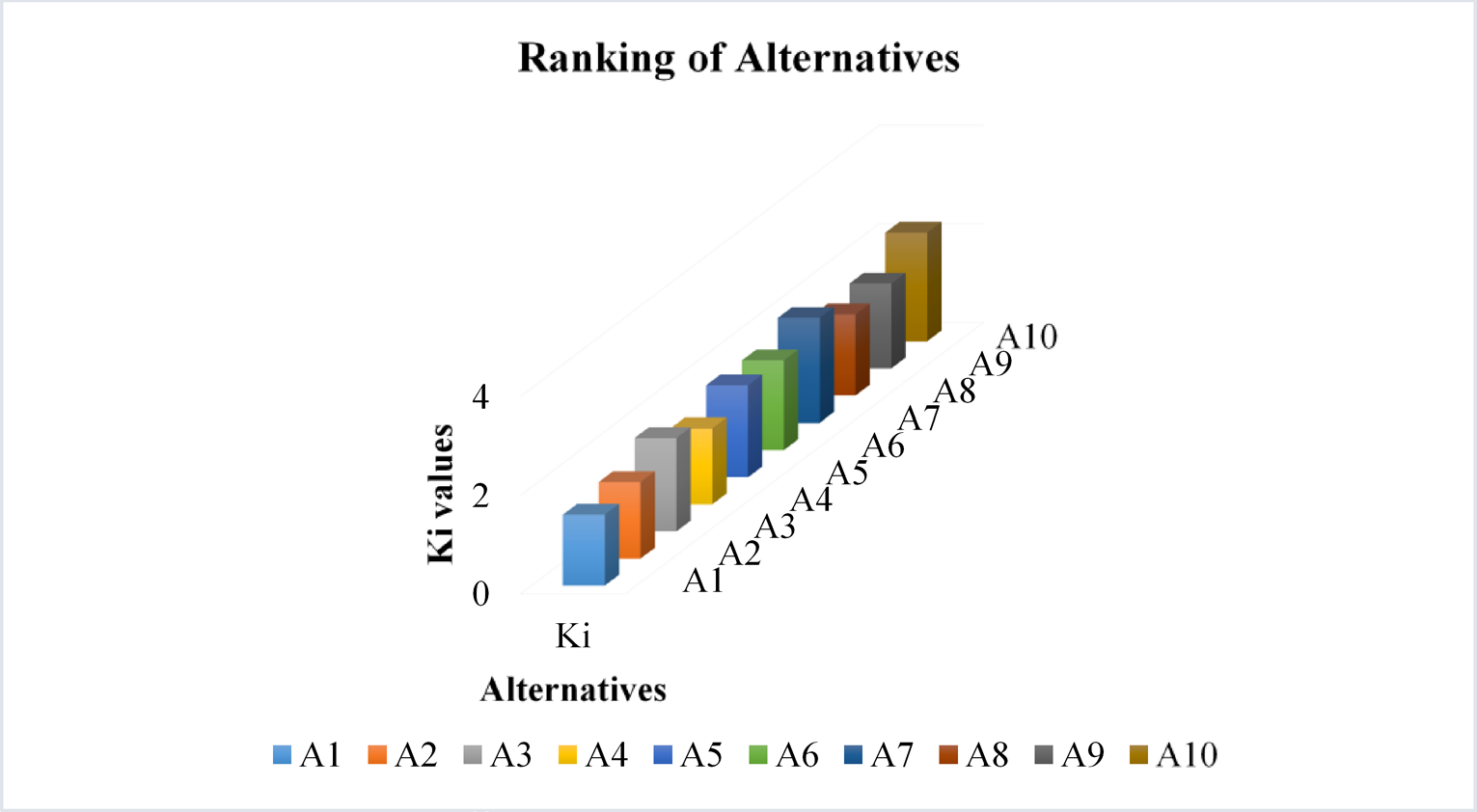
Ranking of alternatives.

The IVTSF-CoCoSo assessment method evaluated martial arts referee selection by evaluating seven prime criteria and ten possible candidates. The ranking system delivers critical perception about referee suitability based on the established decision-making criteria. The scoring process showed$\:\:{R}_{10}$ as the top-ranked alternative because it delivered optimal performance across seven evaluation criteria, thus demonstrating a well-balanced lack of bias, a useful track record, and its ability to make accurate decisions. $\:{R}_{7}$ along with $\:{R}_{3}$ finished second and third respectively because their qualifications were strong, although they performed slightly differently in terms of their ability to maintain consistency and adaptability within stressful situations. Referee $\:{R}_{1}$ demonstrated lower performance than competing officials in crucial aspects that included referee experience, together with decision speed and rule accuracy enforcement. The rankings of $\:{R}_{2}$ and $\:{R}_{4}$ decreased because these referees need to strengthen their abilities in refereeing consistency and technical knowledge. Referees $\:{R}_{6}$, $\:{R}_{9}$, $\:{R}_{8}$ and $\:{R}_{5}$ maintained a balanced performance level yet possessed considerable capabilities in particular areas together with needs for additional development in other aspects. Figure 2 shows the graphical representation of alternatives ranking. The IVTSF-CoCoSo model delivers successful decision-making results through its structured uncertainty-handling system, which results in referees receiving rankings through objective analyses rather than personal biases. The study proves the practical nature of IVTSF-CoCoSo as a referee selection tool by introducing integrated fairness measures that achieve dependable evaluation rankings which support valid tournament choices in martial arts competitions.

## Comparison analysis

To further evaluate the effectiveness of the proposed CoCoSo method, a qualitative comparative analysis has been conducted with traditional MCDM methods, including TOPSIS^8^, VIKOR^9^, SWARA^10^, MOORA^40^, PROMETHEE^11^, and EDAS^41^, as shown in Table 7. This comparison is based on performance indicators derived from existing literature and this study’s application experience. The comparison was based on five criteria critical to sports-related decision-making context: uncertainty handling capability, accuracy in ranking, flexibility in weighting, scalability to large datasets, and domain applicability to referee selection. Each method was rated qualitatively (high, moderate, low) based on the performance demonstrated or limitation in each area. The proposed method exhibits low computational complexity, making it work more efficiently and allowing greater scalability than complex methods such as VIKOR and PROMETHEE. CoCoSo demonstrates better uncertain scenario management than TOPSIS and MOORA in expert ambiguity or vague decision situations. Its superior ranking abilities ensure CoCoSo selects dependable specific referees beyond the moderate achievements achievable with traditional methods, while their ranking systems are adjustable. Weighting choices in CoCoSo perform better than in TOPSIS and MOORA due to its versatile rules framework that changes according to decision frameworks. The method demonstrates great applicability in large datasets since it performs better than SWARA and EDAS methods, which struggle under data scaling needs. CoCoSo stands out as the best choice for selecting referees in martial arts competitions because its implementation supports both data-driven decisions and resolves typical MCDM method weaknesses.

**Table 7:** Comparison analysis.

Criteria	CoCoSo (proposed)	TOPSIS	SWARA	VIKOR	MOORA	PROMETHEE	EDAS
Handling uncertainty	High	Moderate	Low	Moderate	Low	Moderate	Moderate
Ranking accuracy	High	Moderate	Moderate	High	Moderate	High	High
Weighting flexibility	High	Moderate	Moderate	Moderate	Moderate	High	Moderate
Suitability for Large Datasets	High	Moderate	Low	Moderate	Moderate	High	Moderate
Applicability to Sports Decision-Making	High	Moderate	Moderate	Moderate	Moderate	Moderate	Moderate

### Theoretical implications

The study unites CoCoSo algorithm with IVTSFS to advance MCDM theory decision-making methods under uncertain conditions. Multiple barriers hinder the practical implementation of MCDM tools VIKOR and SWARA along with TOPSIS because these tools lack efficient mechanisms to handle complex uncertainty and expert hesitation when processing imprecise input information. Integrating IVTSFS and fuzzy decision-making models improves fuzzy information handling capabilities, thus creating more effective human judgment modeling techniques. CoCoSo and IVTSFS combine an innovative aggregation process that stabilizes ranking results while producing more solid, accurate outcomes. The research establishes innovative ways for advanced fuzzy MCDM models to handle sports decision-making processes. It especially demonstrates their effectiveness in referee selection evaluations which involve dynamic and subjective assessment. Through this investigation, researchers filled the theoretical MCDM advancement gap with applications in practical decision-making, thus establishing groundwork for future research about intelligent decision systems across all domains.

## Sensitivity analysis

The proposed IVTSF-CoCoSo model underwent two independent sensitivity tests, which adjusted parameters $\:t$ and $\:\lambda\:$ to determine its reliability and stability. The specified values of$\:\:t$ and$\:\:\lambda\:$ control uncertainty and weight effects within IVTSFS to determine final referee rankings during selection.

- **Sensitivity analysis by varying** $\:\varvec{t}$

**Fig. 3:**
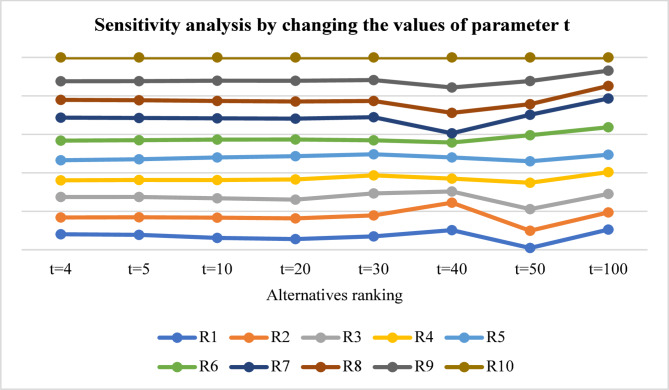
Sensitivity analysis by changing the values of the parameter $\:t$.

The assessment began through changes to the $\:t$ value starting from $\:t=4$ until it reached $\:t=100$ in order to examine IVTSFS uncertainty levels. Figure 3 shows that numerical scores became more variable as $\:t$ values increased but the proposed method maintained its stable ranking structure. The values of $\:t$ at lower ranges resulted in stable rankings, which evolved into minor shifts when values increased, primarily influencing middle-range referees. The results show that the IVTSF-CoCoSo method control uncertainty effectively, therefore maintains consistent decision outputs.

- **Sensitivity analysis by varying** $\:\varvec{\lambda\:}$

**Fig. 4:**
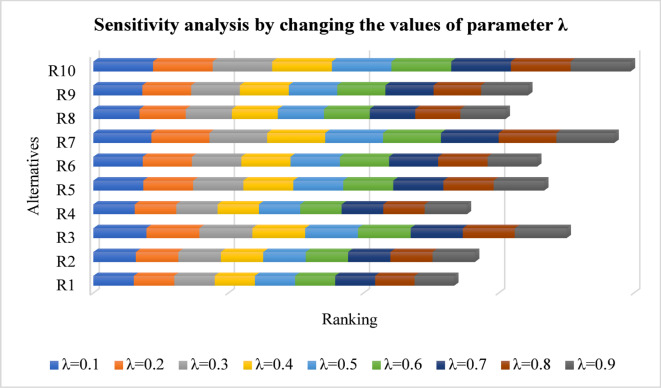
Sensitivity analysis by changing the values of the parameter $\:\lambda\:$.

Figure 4 shows how changes in the $\:\lambda\:$ factor affects weight adjustments during the decision-making procedure. Different values of $\:\lambda\:$ ranging from $\:0.1$ to $\:0.9$ produced minimal alterations in ranking scores that maintained stable overall rankings. The proposed approach demonstrated high consistency through $\:{R}_{10}$ obtaining a stable scoring position regardless of $\:\lambda\:$ variation along with minor score shifts in other referee rankings.

The IVTSF-CoCoSo model stands out due to its robust operational stability and ability to withstand parameter variations according to sensitivity test findings. The combination of $\:t$ and $\:\lambda\:$ parameters showed minimal influence on score outcomes because the calculation system resulted in consistent and dependable position rankings. Through this solution referees can choose reliable referees who provide precise results and high computational efficiency, which provides practical benefits to martial arts competitions.

### Managerial and social implications

The following are the managerial and social implications: The evaluated IVTSF-CoCoSo framework presents a systematic method to select referees because it enables decisions through explicit and mitigates biased selection criteria. IVTSF-CoCoSo provides sports management authorities and tournament organizers with enhanced referee assignment fairness and complete transparency that reduces the influence of human subjectivity during selection. This model provides an effective MCDM procedure with uncertainty handling capabilities, allowing its adoption in other managerial fields, from workforce selection through resource distribution to performance management systems. The approach produces consistent end results through its robust operating abilities that allow better strategic sports governance policies whenever decision variables change. This model strengthens martial arts tournament integrity while building trust between athletes and coaches as well as between all competition participants. Properly mitigating biases in referee assignments throughout sports competitions create inclusive opportunities for officials, decreasing favoritism and discrimination. The deployment of intelligent decision-support systems by sports management organizations can help them create data-based methodologies to amplify AI and fuzzy logic solutions in different social decision systems. Athletes and teams who adopt an objective selection process feel more confident about game results, which strengthens ethical practices and sportsmanship in martial arts, together with other competitive sports. The research enhances sports organizational management through its data-based referee selection system and promotes equality by implementing fair, approachable referral processes. Both uncertainty and vagueness in referee selection processes can be effectively managed through the proposed IVTSF-CoCoSo model for making better decision outcomes.

### Advantages of the study

The advantages of this study are summarized below: The model maintains strong and repeatable rankings throughout different circumstances because of its comprehensive sensitivity analysis that tests significant parameters. The proposed method shows superiority over standard MCDM methods (TOPSIS, SWARA, VIKOR, MOORA, PROMETHEE, and EDAS) regarding ranking dependability, ease of application, and processing time. The study performs referee selection through MCDM with fuzzy logic integration to produce mitigates biases and data-supported objective assessments that enable fair tournament refereeing processes. This practical model enables sports organizations as well as tournament controllers to generate proper referee assignments through systematic decision-making processes. The proposed model demonstrates potential for application in additional domains such as human resource assessment, sports performance outlook and financial choice determination. The technique efficiently manages intricate decision tasks without creating significant computational issues that work well for large-scale programs. The research benefits demonstrate the important value of the technique, which establishes it as an essential advancement in decision science, sports management, and MCDM research fields.

### Limitations

The IVTSF-CoCoSo model achieves high accuracy together with stability features and reduces computational requirements yet it faces particular implementation barriers that require improvement. The decision-making process reaches better quality outcomes due to weighting criteria but becomes more subjective through expert judgment application. Fuzzy logic implementation within this model addresses subjectivity because it can detect and handle unclear situations effectively. This specific martial arts referee selection application showcases domain accuracy preservation within the model while showing its potential for use in human resource management decisions, financial decision-making, and industrial assessments. Different aspects of IVTSFS operation may seem challenging initially, however, they represent high-level uncertainty in a manner that outperforms traditional MCDM approaches.

Although the current research proposes a referee ranking model using technical, psychological, and communicative decisive criteria, operational realities influencing ultimate referee appointment must be recognized. These are geographical accessibility, travel restrictions, time restrictions, and the moral spread of opportunities to referees. As an illustration, giving several events to the highest-ranked referee regardless of the rest time or rotation fairness would result in workforce dissatisfaction and a long-term decrease in organizational resilience. So, the existing IVTSF-CoCoSo model can be considered a fundamental assessment device, which helps decision-makers to select the most competent candidates. The final assignments should include additional constraints that can be handled by scheduling software, rules-based policy, or human intervention to ensure balanced and sustainable referee use. This tiered solution can reduce unintended consequences without losing the advantages of the model, which entail fairness, transparency, and data-driven decision-making.

## Conclusion

This paper proposed an effective and formalized referee selection model in martial arts competition via a combination of the IVTSFS and CoCoSo MCDM methodology. The selection of referees in martial arts has traditionally been associated with manual selection by a committee member or senior referee. In most cases, it may involve subjective analysis, personal opinion, or a lack of continuity in evaluation standards. Such traditional methods are not transparent, inconsistent, and do not allow measuring the uncertainty of expert opinion. The IVTSF-CoCoSo approach proposed in this paper deals with such issues by allowing decision-makers to communicate their judgments regarding interval-valued and hesitation-enriched fuzzy assessments. This enables the system to realistically model expert uncertainty, emotional hesitation, and multi-dimensional judgement across criteria, e.g., experience, correctness of decision, rule knowledge, and stress management. It showed that $\:{R}_{10}$ was the best referee candidate, having the highest overall performance according to the established criteria, followed by $\:{R}_{3}$ and $\:{R}_{7}$, and the least performer was $\:{R}_{1}$. The validity of this ranking was also confirmed by comparison with other classical MCDM techniques, in which the proposed method was shown to perform better in terms of uncertainty treatment, ranking quality, flexibility of the weighting scheme, and applicability to large-sized datasets. The final rankings were stable, and a sensitive analysis supported the finding by using different weights in the decisions. The paper presents a decision-support system that increases the referee selection in martial arts contests regarding fairness, transparency, and reliability. The IVTSF-CoCoSo model enables sports federations, tournament directors, and officiating committees to leave the subjective assessment behind and follow a more objective, evidence-based, data-driven process of evaluating referees. The model’s flexibility implies that it could be used in a broader range of sports-management decisions involving uncertainty.

The future scope should investigate this model using alternative interconnected fuzzy frameworks which unite SFS^42^ with interval-valued PFS^43^, complex PFS^44^, and bipolar hesitant fuzzy sets^45^ to enhance decision outcomes. The aggregation accuracy can increase using Maclaurin symmetric mean operators^46^ alongside Muirhead mean operators^47^. The methodology functions as a tool that medical diagnosis experts like^48,49^ applies it for disease risk evaluation together with other MCDM problems to increase universal applicability and structural reliability.

## Data Availability

The datasets used and/or analyzed during the current study are available from the corresponding author upon reasonable request.
